# Assessment of Registration Methods for Thermal Infrared and Visible Images for Diabetic Foot Monitoring

**DOI:** 10.3390/s21072264

**Published:** 2021-03-24

**Authors:** Sara González-Pérez, Daniel Perea Ström, Natalia Arteaga-Marrero, Carlos Luque, Ignacio Sidrach-Cardona, Enrique Villa, Juan Ruiz-Alzola

**Affiliations:** 1 Industrial Engineering Department, Faculty of Engineering and Technology, University of La Laguna, 38200 San Cristóbal de La Laguna, Spain; 2 IACTEC Medical Technology Group, Instituto de Astrofísica de Canarias (IAC), 38205 San Cristóbal de La Laguna, Spain; narteaga@iac.es (N.A.-M.); carlos.luque@iac.es (C.L.); ignacio.sidrach@iac.es (I.S.-C.); evilla@iac.es (E.V.); juan.ruiz@ulpgc.es (J.R.-A.); 3 Computer Science and Systems Department, University of La Laguna, 38200 San Cristóbal de La Laguna, Spain; dani@isaatc.ull.es; 4 Research Institute of Biomedical and Health Sciences (IUIBS), Universidad de Las Palmas de Gran Canaria, 35016 Las Palmas de Gran Canaria, Spain

**Keywords:** registration, medical imaging, thermography, diabetic foot, ICP, homography, ASGD

## Abstract

This work presents a revision of four different registration methods for thermal infrared and visible images captured by a camera-based prototype for the remote monitoring of diabetic foot. This prototype uses low cost and off-the-shelf available sensors in thermal infrared and visible spectra. Four different methods (Geometric Optical Translation, Homography, Iterative Closest Point, and Affine transform with Gradient Descent) have been implemented and analyzed for the registration of images obtained from both sensors. All four algorithms’ performances were evaluated using the Simultaneous Truth and Performance Level Estimation (STAPLE) together with several overlap benchmarks as the Dice coefficient and the Jaccard index. The performance of the four methods has been analyzed with the subject at a fixed focal plane and also in the vicinity of this plane. The four registration algorithms provide suitable results both at the focal plane as well as outside of it within 50 mm margin. The obtained Dice coefficients are greater than 0.950 in all scenarios, well within the margins required for the application at hand. A discussion of the obtained results under different distances is presented along with an evaluation of its robustness under changing conditions.

## 1. Introduction

Diabetes is a chronic disease that affected 463 million adults (20–79 years) in 2019 according to the International Diabetes Federation (IDF) [1] and this number was expected to rise to 700 million by 2045. Peripheral neuropathy is the most common form of diabetic neuropathy, affecting the outer nerves of the limbs, particularly those of the feet, and contributes to the appearance of ulcers that can lead to lower limb amputations when not treated in time [2,3,4]. Diabetic foot is one of the most common, costly, and severe complications of diabetes that especially affects low-income communities [1]. A strong correlation between the severity of the neuropathy and the skin hardness has been reported in diabetic patients [5,6]. Moreover, detection of an anomalous superficial temperature can be indicative of an ulcer formation in an early stage. Therefore, screening methods based on foot temperature detection have been identified as leading technologies in this field [7,8,9,10]. The specialized technology to monitor these symptoms involve a time-consuming procedure that traditionally uses contact sensors, and as a consequence most of the cases are detected when damage is already done [11,12,13,14,15]. A clinically effective monitoring protocol for diabetic foot ulcers requires comparing the temperature of contralateral-matched plantar locations on a daily basis, but this can be an expensive, time-consuming procedure that is difficult to maintain in a self-monitoring scenario [4,8]. Therefore, a fast and precise screening system to measure plantar temperature is needed to improve this screening protocol.

Low cost and off-the-shelf thermal and RGB cameras have been integrated in various prototypes designed for the remote monitoring of diabetic foot neuropathies, in order to detect anomalous temperature patterns in the subject’s feet that are an early indicator of an ulcer formation [16,17]. The increase in the availability of affordable off-the-shelf thermal cameras (Thermal Expert, Seek, FLIR) in combination with the already inexpensive RGB cameras allows for the design of new low cost configurations that usually confront multiple barriers in order to be usable for different applications. One of these barriers is the synchronization of different sources when this is not possible via hardware (e.g., an external trigger) [18,19]. RGB and IR images will not be spatially registered since the optical characteristics of the sensors and their relative position and orientation differ from one another. This will be another barrier to overcome since the lack of information between RGB and thermal cameras makes it difficult to perform an extrinsic calibration using typical techniques [20,21,22,23,24].

The achievement of a correct registration and fusion of two different sources will strongly depend on the application, so the registration method applied can differ in terms of complexity, computation time, usability, and robustness. Research on registration and fusion algorithms in theory and application has developed rapidly in recent years and some of these approaches study the case of integration of visible and IR sensors for different applications [22,25,26,27,28,29]. Some studies have addressed the case of the application of remote monitoring of diabetic foot, but the registration strategies are mostly constrained to methods that show limitations or low performance. Liu et al. [30] apply a rigid transformation in order to correct the distortions introduced by the subject distance but an analysis on the performance degradation with distance together with other transformation methods is not carried out. Other studies focus only on the thermal images, and do not perform registration between thermal and visible spectrum images, limiting the registration to contralateral thermal images comparisons, thus not confronting the challenges of using heterogeneous sensors and its matching [31,32]. To the best of the authors’ knowledge, a consistent evaluation of different registration methods in order to assess the best choice for the diabetic foot monitoring application has not yet been carried out.

A consistent and accurate registration method between visible and thermal infrared images is a prerequisite to obtain a multispectral image for abnormal plantar temperature detection, where both visible and IR spectra are used together. Once the registration process has been successfully achieved, other phases of the analysis can be carried out. These phases may include the extraction of the areas of interest by segmenting the sole of the feet to detect temperature deviations via contralateral thermal comparisons, IR-visible mapping between temperature deviations and visible ulcer damage, and dataset generation of multispectral feet images where each pixel has the information of all the channels aligned. The presented work is focused on the registration process as a necessary step within the diabetic foot diagnosis protocol. Subsequent steps in the protocol are out of the scope of this contribution and have been discussed in previous publications [16,17].

In this contribution, we analyze four different registration methods to obtain the fusion of visible and thermal infrared images obtained with low cost cameras to be used for a medical application: early detection of diabetic foot neuropathies [1]. Firstly, we describe the prototype characteristics, the image acquisition process, and the technique used to carry out the synchronization of the integrated cameras. Secondly, we introduce the sensors calibration process along with the four image registration methods. Then, the image registration and fusion methods are evaluated using quantitative metrics and qualitative criteria, both when the subject’s feet are located at the focal plane distance of the cameras and outside of it (within 50 mm margin). Finally, the advantages and disadvantages of all registration methods are discussed followed by an evaluation of the best performing method to be implemented in the diabetic foot prototype.

## 2. Materials and Methods

### 2.1. Image Acquisition

The prototype for remote monitoring of diabetic foot has two sensors, a RGB camera in the visible spectrum and a microbolometer sensor in the thermal infrared spectrum. An Intel^®^ RealSense™. D415 camera (Intel Corporation, Santa Clara, CA, USA) was used to acquire the images in the visible spectrum, which includes red, green, and blue color information plus depth measurements (RGB-D). A low cost thermal camera model TE-Q1 Plus from Thermal Expert™ (i3system Inc, Korea) was used to acquire images in the thermal infrared (IR) spectrum. The resolution of the RGB-D images is 1280 × 720 pixels and the IR images were acquired with a resolution of 384 × 288 pixels, i.e., the maximum Thermal Expert™ TE-Q1 Plus sensor resolution. These cameras, previously characterized and calibrated, were found to be suitable for the presented medical application [15]. Both cameras were mounted in a customized support that kept them aligned and with a fixed relative position and focus. In order to obtain useful information of the sole of the feet, this custom acquisition system requires calibration, synchronization, and registration among different imaging sources (RGB-D, IR) [16,17].

For each sensor, six images were acquired at varying working distances between the cameras and the feet, ranging from 760 mm to 850 mm. Notice that the optimal distance would be 800 mm, as this is the distance between the sensors and the fixed focal plane. In order to analyze the quality of the results for different distances around the focal plane, a total of six positions were analyzed, moving the feet forward and backwards from the focal plane distance. Apart from the variable working distance, no additional constraining protocol was considered for the image acquisition. These images were manually segmented by two independent researchers with the aim to extract the sole of the feet from the background. The segmentation was performed in both IR and visible images employing the software application ITKSnap [33]. Subsequently, the segmentations from each researcher were merged using a probabilistic estimate of the true segmentation for each image employing the Simultaneous Truth and Performance Level Estimation (STAPLE) algorithm [34]. The output is the set of reference segmentations to be used as input for all the analyzed registration methods.

#### 2.1.1. Synchronization

The cameras used in this study operate at different image frame rates (frames per second, fps). This is a problem for image registration because a scene is captured at different points in time. To solve this issue, a camera sync procedure was implemented in the workflow of image acquisition of the diabetic foot application.

Camera synchronization can be done both at hardware and software level. At the hardware level, in an instant, one or more hardware signals are activated and sent to the cameras to tell them to proceed to capture the images. At the software level, one or more software events are generated in each camera software development kit to receive the images.

The thermal camera selected for this application does not allow for synchronization via hardware, so the capture workflow of both cameras were analyzed in order to implement a software technique for camera synchronization. A combination of various software synchronization techniques was carried out, based on the application of a timestamp (using the system time) at image reception to every image. The implemented technique assigns a reception timestamp to each image and stores each image in a buffer according to the type of camera. This process is activated by a software event and is deactivated after a fixed amount of time, which in our case is two seconds. Therefore, two seconds of images are stored for each camera type in buffers to minimize the number of images in temporary memory. Then, the two images of each type with the smallest time difference between them are selected. These two images are considered synchronized and suitable for the application at hand where the subject movement is negligible.

#### 2.1.2. Calibration

**Figure 1:**
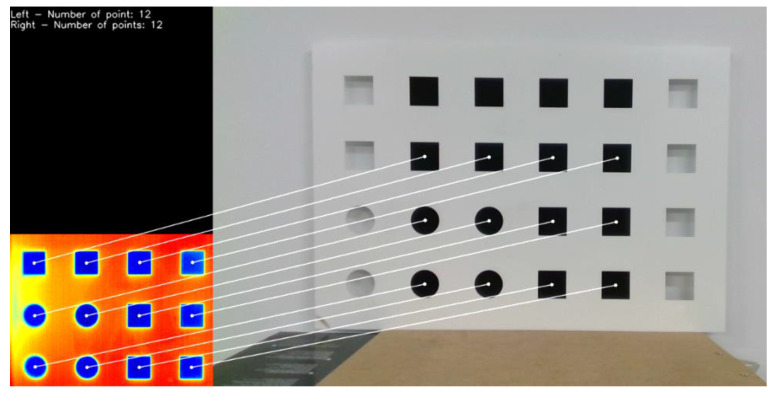
Custom built checkerboard keypoint detection and matching. (**Left**) Thermal image with the keypoints detected in the features of the checkerboard. (**Right**) RGB image with the keypoints detected in the features of the checkerboard. White lines show the corresponding thermal and visible keypoint pair. Notice the black aluminum plate behind the checkerboard in the RGB image and its corresponding cold contrast signature in the thermal image.

In order to be able to register the images obtained with the visible and IR cameras, we need to calculate the calibration parameters of each sensor and the relative pose between them. Our registration methods allow the capture of both sets of parameters using visual keypoints within each image. Some registration methods, like the Geometric and Optical Translation and the Homography applied in this study, use an image calibration checkerboard where the target keypoints and all their relative positions and dimensions are known. This checkerboard-based calibration uses an object with known dimensions and stable keypoints to find corresponding points in the camera images [20]. This technique is based on a widely accepted calibration method proposed by Zhang [23]. In this application where visible and IR sensors are in use, there is a low correlation between the perceived visible and thermal infrared spectrum features. Therefore, a special checkerboard was designed in order to achieve both the visible and heat signature keypoints (see Figure 1). For this study, we designed a PVC checkerboard with a laser plotter, made of squares and circles with a black aluminum sheet that is located in the back. This aluminum sheet is cooled down in a fridge in order to obtain a well-defined heat contrast in the thermal image between the squares and circles of the PVC checkerboard and the background sheet.

### 2.2. Image Registration

The registration methods applied throughout this contribution aim to establish the registration transformation between the thermal infrared and visible spectrum images. The extracted transformation matrices establish the mathematical model to transfer the coordinate system of images acquired with one sensor towards the other (or vice versa). These depend solely on the optical characteristics of the sensors and their relative position and orientation, which are fixed in our acquisition system. Therefore, the transformation matrices are not dependent on the subjects’ feet properties. Once established, they are valid without a need of recalibration between subjects, maintaining the registration performance across acquisitions as soon as normal operational margins are kept. The chosen registration methods are representative of transformation models in the image processing literature based on the numbers of degrees of freedom that the model supports, e.g., scaling, translation, rotation, sheer, perspective correction, as well as based on the mechanism of extraction of the transformation matrices, i.e., feature-based or intensity-based.

#### 2.2.1. Geometric Optical Translation (GOT)

The first registration method uses a geometric approach to obtain the registration of the images provided by both visible and IR cameras. First, the Field of View (FOV) and angular resolution of the visible and IR cameras were obtained using the intrinsic optical parameters and the specifications of both sensors provided by the manufacturers. This information was used to match the angular resolution of each sensor’s images via a simple scaling operation. The visible images were translated to match the coordinate system of the IR camera using a translation transformation defined as:

(1) $$T_{\mu }\left(x\right)\;=\;x+t$$

The translation vector and the parameter vector of the transformation is directly defined by μ=t The values of the parameter vector μ were obtained finding the relative positions of several checkerboard keypoints in each sensors’ coordinate system via visual inspection. Finally, a cropping procedure was included to match the coordinate system and the resolution of the thermal images.

#### 2.2.2. Homography

The second registration method allows for the greatest flexibility relying on a transformation with the largest amount of free parameters, and is defined by the Homography transformation:

(2) $$T_{\mu }\left(x\right)\;=\;H\left(x\right)$$

In order to get these extra degrees of freedom, the transformation is defined by the full homography matrix H with no restrictions so the image can be translated, rotated, scaled, sheared, and perspective corrected. The parameter vector *µ* is formed by the H matrix elements, obtaining a total of eight parameters. To obtain the optimal transformation parameter vector *µ* in this large search space, we rely on the constraints imposed by the keypoints provided by the calibration checkerboard, knowing that their distribution and relative positions within each image is known upfront. The keypoints detection is performed by applying a standard blob feature detector over the images, and then pairing the set of keypoints of one image with the corresponding counterpart in the other image (see Figure 1). Once the pairing is obtained, we find the set of parameters values that satisfy an homography transformation via a RANSAC [35] statistical outlier removal process, finding the best set of keypoints that map one image to the other and removing outliers that do not support the transformation.

In this application, we used the implementation of the blob feature detector and the Homography RANSAC solver available in the OpenCV software library [36].

#### 2.2.3. Iterative Closest Point (ICP)

The Iterative Closest Point (ICP) algorithm is a method for aligning two sets of arbitrary points and was first introduced by Besl and McKay [37]. This method uses a rigid transform $T_{\mu }$ that can be defined as:

(3) $$T_{\mu }\left(x\right)\;=\;R\left(x-c\right)+t+c$$

where the parameter vector is defined by μ=t, R is the rotation matrix (i.e., orthonormal and proper), c the center of rotation, and t the translation applied. The image subject to the transformation is considered as a rigid body, and will be translated and rotated by the algorithm without allowing for scale changes during the registration process. Any difference in scale between the two images is handled by a fixed scaling ratio previously obtained (see Section 2.2.1). The rotation matrix is parameterized by Euler angles. The center of rotation has been fixed at the center of the image.

This method uses the rigid transformation in order to align a set of keypoints previously obtained from both input images using an edge detection algorithm. The goal of ICP is to compute a rotation matrix R, and a translation vector t, so that the transformed data points are best aligned with each other, minimizing the Root-Mean-Squared-Error function (RMSE) where the errors are the distances between each pair of keypoints.

A relevant difference of this method versus the previous ones is that the keypoints used to do the registration are obtained from the image inputs directly rather than using the checkerboard features from the calibration process. The input pair of images in this case are the mask images that contain the segmented feet in the visible and thermal spectra respectively. In order to obtain the keypoints, the input images are processed with an edge detection filter to extract the contours of the objects in the image (in our case, the subject’s feet). Finally, a predefined number of points are extracted from the contours to be used as the keypoints set to be matched in the iterative registration process.

#### 2.2.4. Affine Transformation with Gradient Descent (Affine-ASGD)

The last registration method allows for greater flexibility than the ICP method, relying on a transformation with more free parameters, and it is defined as an affine transformation expressed as:

(4) $$T_{\mu }\left(x\right)\;=\;A\left(x-c\right)+t+c$$

In order to get these extra degrees of freedom, the transformation matrix A has no restrictions so the image can be translated, rotated, scaled, and sheared. The parameter vector *µ* is formed by the matrix elements $a_{ij}$ and the translation vector t, obtaining a total of six parameters. To obtain the optimal transformation parameter vector *µ* in a search space with such a large number of dimensions, a sampling-based optimization algorithm was used to shorten the processing time. The chosen algorithm is the adaptive stochastic version of the gradient descent optimization algorithm (ASGD) developed by Klein et al. [38], that introduces multiple speedups in the convergence of the minimization process, while reducing the number of parameters that controls the algorithm [39]. This strategy often improves convergence performance over standard stochastic gradient descent in settings like image recognition and processing, as in our current application. Notice that the stochastic nature of the sampling process introduces a relevant difference between this method and the ICP method, in the sense that its results are not deterministic anymore under the same set of inputs, while ICP keeps a deterministic result on every run. In this application, we used the implementation of ASGD available in the Elastix software library [39,40].

## 3. Results

In order to assess the best approach for registering the visible and the thermal infrared images of the diabetic foot application, 12 images (6 visible and 6 IR) were analyzed using four registration methods (see Section 2). These images were taken with the subject’s feet at the chosen focal plane distance for the sensors (800 mm) and at different distances backward and forward from this plane, in order to evaluate the robustness of the registration method under small changes in the position of the subject’s feet with respect to the sensors. The obtained results are shown in the next sections, starting with an analysis of the results at 800 mm, and following with the analysis for different positions without changing the optical parameters of the sensors.

To evaluate the performance of the registration algorithms, we used the reference segmentations (STAPLE) of the patient feet to measure the final overlap of the registered images. The chosen performance metrics are the Dice coefficient and the Jaccard Index (Intersection over Union, IoU) of the segmentation masks, being two of the most commonly used metrics in semantic segmentation [41,42]. Moreover, we also calculated other performance metrics such as the false positives (Specificity) and false negatives (Sensitivity) using the thermal images segmentation as the reference source [43,44].

### 3.1. Performance at 800 mm (Focal Plane)

**Figure 2:**
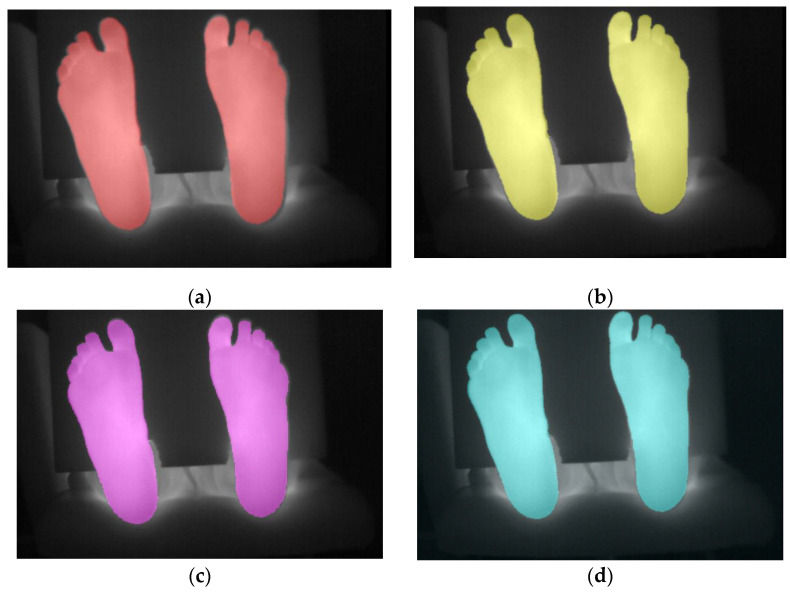
Fusion of thermal IR images with the feet segmentation of the visible image after applying each registration method (masks highlighted in color) analyzed at 800 mm. (**a**) GOT, (**b**) Homography, (**c**) ICP, and (**d**) Affine-ASGD registration methods.

**Figure 3:**
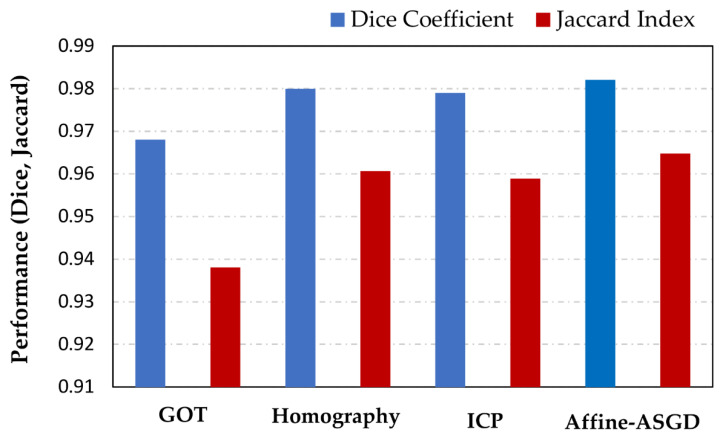
Dice coefficient and Jaccard index metrics for the four registration methods obtained with the subject located at the sensors focal plane (800 mm).

The fusion of the thermal images and the registered visible masks, obtained with the different registration methods when the patient feet are located at the focal plane distance, is shown in Figure 2. It can be seen that all methods obtain a visually correct overlap between the original thermal image and the registered visible masks, when the subject is located at the focal plane distance of the cameras (800 mm). The correct alignment of the visual image mask and the original thermal image confirms the suitability of the methods for this application under this restricted condition. The performance of these four methods in terms of the Dice coefficient and Jaccard index is displayed in Figure 3.

#### 3.1.1. Geometric Optical Translation (GOT)

The results indicate that using just the geometric and optical information to obtain the simplest registration method of IR and visible images delivers the lowest performing results, both using the Dice coefficient (1.4% less than the best method) and Jaccard index (see Figure 3). This method simply applies axis-aligned translations between the two sensors, thus any rotation or optical distortion difference between the two sensors will not be compensated in the registration process. However, this method shows a good performance (Dice coefficient = 0.968) when the subject is located at the focal plane distance of the cameras (800 mm).

#### 3.1.2. Homography

The results obtained from the Homography method are shown in Figure 2 and Figure 3. The results obtained when the subject feet are located at the focal plane distance (800 mm) are correct with a Dice coefficient of 0.980, and are comparable to the results obtained with the GOT method albeit improving them slightly (1.2% better).

The Homography transformation has more degrees of freedom than any other method used in this study. It performs scaling, translation, rotation, and perspective correction of the images, but to do so, it only uses the keypoints that implement the mapping between both images, obtained from the calibration images with the custom checkerboard. This mapping limits the output quality, as the registration performance is strongly coupled with the accuracy of the calibration checkerboard and its keypoint detection process. The perspective correction will be as good as the quality of the detection of these reference points without errors, especially in the thermal image, becoming the main limitation for the quality of the registration. By having more degrees of freedom, any noise that we have at the inputs is going to affect the results, showing up as an increased error in the segmentation overlap.

#### 3.1.3. Iterative Closest Point (ICP)

The Iterative Closest Point method also obtains correct results for the registration of the images captured with the subject’s feet located at the focal plane distance (800 mm), similarly to the previous methods (Dice coefficient = 0.979) as shown in Figure 2 and Figure 3. These results are slightly lower than the Homography method at the focal plane (0.1% less), which is reasonable given that the transformation applied by ICP has less degrees of freedom than the Homography, namely not able to apply perspective corrections. Nonetheless, the results are still competitive, exploiting the information contained in the input images themselves rather than being limited by the number of keypoints present in the calibration checkerboard. This method also has the advantage that it does not require the usage of the checkerboard calibration images to obtain the transformation, simplifying the overall process.

**Figure 4:**
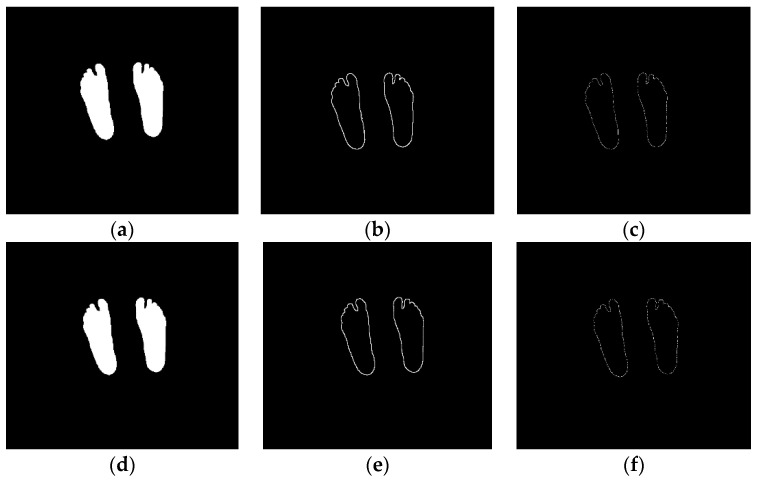
ICP image processing pipeline to extract the set of keypoints. Left: input images used for this application, (**a**) visible STAPLE mask image and (**d**) thermal STAPLE mask image. Center: edge detection filtering result that extracts the feet contours, (**b**) visible edge detection and (**e**) thermal edge detection. Right: keypoints selection used for the ICP registration process, (**c**) visible mask keypoints used for ICP and (**f**) thermal mask keypoints used for ICP.

As we can see in Figure 4, the visible and thermal masks (STAPLE) obtained at 800 mm from the sensors are the input to the ICP registration process, starting with an edge detection filter applied to each mask and obtaining the feet contours. Finally, a predefined number of keypoints is extracted in order to carry out the iterative closest point algorithm. This algorithm converges the registration to the closest transformation that minimizes the distance between the points over a set of iterations.

#### 3.1.4. Affine Transformation with Gradient Descent (Affine-ASGD)

As shown in Figure 2 and Figure 3, the Affine transformation method obtains the best result (Dice coefficient = 0.982) for the registration of the images captured with the subject’s feet located at the focal plane distance (800 mm), slightly better than the Homography method (0.2% better). The improvement over the other methods is expected given that it uses a combination of a reasonable number of degrees of freedom together with a direct keypoint extraction from the input images and an iterative minimization process.

**Figure 5:**
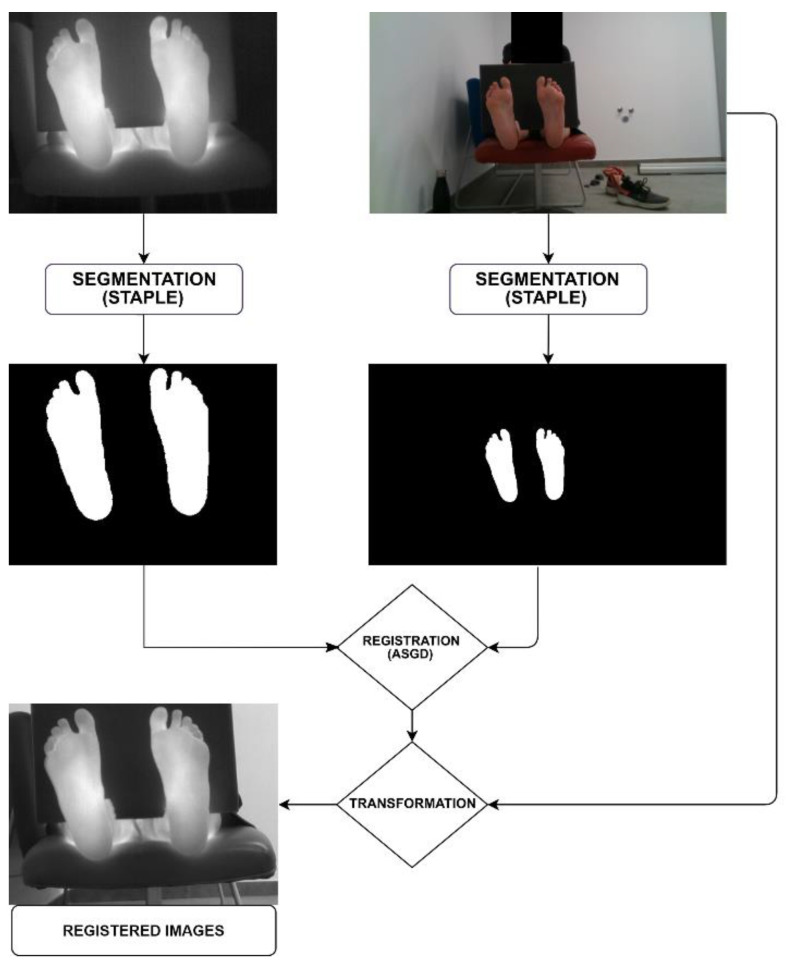
Affine-ASGD method registration pipeline.

In our application, the (STAPLE) masks of the feet obtained for both the original visible and thermal images are used as inputs for finding the best affine transformation via the ASGD algorithm, taking the thermal mask as the reference image, and finding the transformation to be applied to the visible mask. Once the best transformation is obtained after the iterative process, the associated scaling, rotation, translation, and sheer is applied to the visible image mask to register it to the thermal image (see Figure 5), as in previous methods.

### 3.2. Performance Comparison of 4 Methods at 800 mm (Focal Plane)

All four registration methods obtain a visually correct overlap between the original thermal image and the registered visible masks when the subject is located at the focal plane distance of the cameras (800 mm). Figure 3 shows the GOT method as the worst performing one for the 800 mm subject’s feet distance. The Homography method performs better than GOT in this specific case (focal plane), and the ICP method performs similarly to the Homography. Finally, Figure 3 shows the Affine-ASGD method performing better than the other three methods (GOT, Homography, ICP) for 800 mm subject’s feet distance.

### 3.3. Performance with Distance (Four Different Methods)

**Figure 6:**
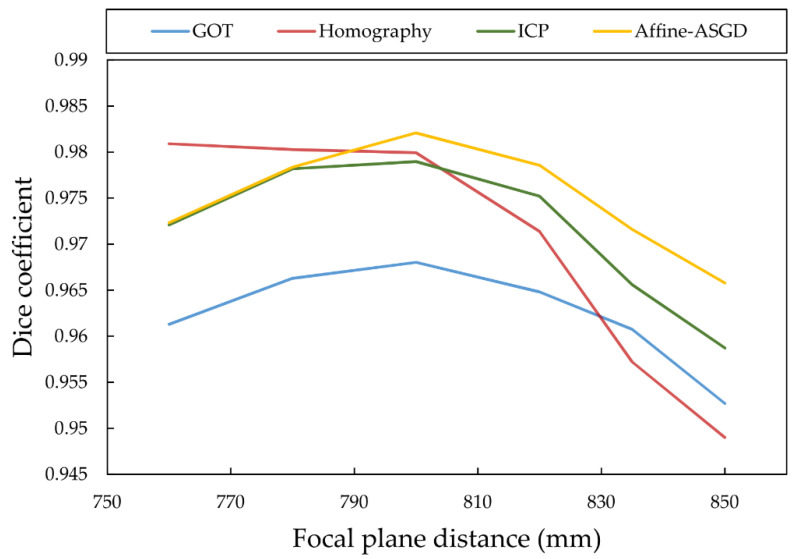
Dice coefficient metric with varying patient feet distance for each registration method.

In order to quantify the robustness of these registration approaches, the transformation values obtained at the focal plane distance (800 mm from the sensors) were also applied to the images acquired at different distances (760 mm, 780 mm, 820 mm, 835 mm, and 850 mm). The complete performance results of the four methods at all different distances are detailed in Table 1. Specifically, the Dice coefficient measurements for the four methods are shown in Figure 6. The results show that the registration tends to deteriorate at distances outside the reference 800 mm mark.

Overall, Figure 6 shows that the performance of all registration methods deteriorates as the subject’s feet distance to the sensors deviates from the focal plane, but this decrease in performance is more significant when the subject is located further away from the sensors. The Dice coefficient degradation is not so pronounced when the distance between the subject and the sensor decreases, and this is explained by two factors. First, the increase in the number of pixels in the feet mask for both images under the same registration deviation makes the Dice coefficient to increase, as the numerator of the coefficient quotient is defined as twice the number of pixels of the intersection of each mask, that grows faster than the number of pixels that do not overlap (denominator of the quotient) when the registration is already converged. The second factor is the better segmentation of the feet performed on the images when the feet is closer to the sensor, given that there are more pixels dedicated to the feet within the image.

## 4. Discussion

Feature-based and intensity-based registration methods have been implemented and evaluated to match thermal infrared and visible images for a diabetic foot application. The registration procedure must ensure that the images obtained from different sources become completely aligned since any deviation between the acquired multispectral images will directly impact the accuracy of abnormal plantar temperature detection.

Analyzing the obtained results with the subject’s feet located at the focal plane distance of 800 mm from the sensors, the GOT method has the lower performance results (see Table 1). This result is explained by the limited number of degrees of freedom of the GOT method, which constrains the maximum quality that can be attained in the registration process. It also relies on the keypoints extracted from the checkerboard calibration images, limiting the accuracy of the registration mapping to fewer data points and increasing the impact of the keypoint detection errors into the registration results.

The Homography method is much more unconstrained, having the largest amount of degrees of freedom of all four applied methods. This greater adaptability can be seen in the improved results obtained when comparing it with the GOT method. Still, the Homography method relies on the keypoints detected in the checkerboard images, inheriting some of the limitations of the process as the GOT method.

The ICP method also has a larger amount of degrees of freedom than the GOT method, but it is limited to rigid transformations, not allowing for perspective corrections. On the other side, it uses keypoints obtained from the input images directly, getting a richer source of information, as the number of visual features is much larger than in the checkerboard calibration images. These properties show up in the better results obtained when comparing it with the GOT method. The ICP method obtains similar results as the ones obtained with the Homography method, but it benefits from a greater robustness on the scenarios where the subject is located at larger distances from the sensor.

The Affine-ASGD method has a large amount of degrees of freedom, namely it allows for scale, rotation, translation, and sheer transformations. This method also uses visual information obtained from the input images, inheriting some of the advantages that also apply to the ICP method. These properties are reflected in an overall improvement of the registration performance when compared to all other methods analyzed in this study.

Analyzing the results from the perspective of the degrees of freedom of the underlying transformations, the registration performance is not directly related with this number. The Homography method, although it has the largest amount of degrees of freedom and it obtains good results for the reference distance of 800 mm, its transformation performance degrades faster than the other methods, showing that it does not generalize well outside of the reference images. This result supports the conclusion that having more degrees of freedom may seem as an advantage, but can also make the algorithm try to over fit the free parameters to compensate for errors that are not modelled by the transformation model itself, e.g., non-linear distortions in the optics. This is true in the case of the Homography method that relies on a linear transformation model with a larger number of degrees of freedom.

From a general perspective, the registration methods that rely on the checkerboard keypoints (GOT and Homography methods) have their performance limited under different distances and optical variations, given that the checkerboard calibration images were obtained at the focal plane of 800 mm from the sensors. Thus, any variation from this reference plane will translate into worse registration results.

The methods that do not use the checkerboard keypoints in the registration process (ICP and Affine-ASGD) are also affected by the distance, but the cause comes from a different source. In the current application where each pair of images come from different light wavelengths (visible vs. thermal infrared), the visual features are not directly comparable and require pre-processing. In this case, the input images to each method are the segmentations performed offline via the STAPLE approach at the reference distance of the focal plane; thus, the obtained transformation is defined for that distance and is not robust to differences where the subject is closer or further away from the reference of 800 mm. Nonetheless, the results are better overall than with the methods that rely on the checkerboard keypoints, as there is much more information extracted from the images themselves that allow for a better extraction of keypoints, rather than being limited to the keypoints in the checkerboard and the quality of its detection process. Moreover, these methods have the advantage of not requiring the usage of the checkerboard calibration images to obtain the transformation, allowing the usage of these methods even after slight changes in the camera poses or optical properties that may happen during the lifetime of the capture device prototype.

While there is a possibility of applying the ICP and Affine-ASGD methods online directly to the original sensors images, the convergence of the minimization algorithms is far from guaranteed, given the large difference in the visual features perceived by the sensors of different spectra. Nevertheless, this drawback can be overcome by applying an automatic segmentation process in the pipeline, enabling the online usage of these methods at the image capture time obtaining robust results, even with subject’s distance or optical parameter changes within reasonable margins. In the case of the ICP method, an online version could be developed using the transformation values of a method for fixed distances, as the initial transformation guess for the online method. The ICP method can converge with this initialization; meanwhile, the difference in the final transformation to be obtained does not differ significantly from the initial guess, allowing the optimization process to converge to the correct solution without being stuck in local minima. This is possible as the ICP method extracts the visual features via edge detection. The Affine-ASGD method is not capable of converging correctly with images from sensors of different spectrums, given that ASGD relies on similarities at the pixel level rather than on visual features. This difference gives an advantage to the ICP method, making it suitable to be implemented in an online fashion in future work.

## 5. Conclusions

The diagnosis of diabetic foot at an early stage requires a fast and contactless procedure with devices that simplify and reduce the cost of the protocol. However, in order to achieve this goal, the screening method and protocol should be implemented, maintaining a high level of accuracy at the diagnosis, ensuring that data acquisition and processing do not introduce systematic errors that can be avoided at design time. Medical diagnosis devices for diabetic foot that combine images of multiple wavelengths require the correct alignment of the obtained data, as any deviation between the acquired multispectral images will directly impact the accuracy of the subsequent abnormal plantar temperature detection. The performance assessment of the registration techniques used at the core of this alignment is key to guarantee a successful monitoring protocol.

Four registration methods have been evaluated to match thermal infrared and visual images from a subject’s feet. All registration methods perform correctly for the application at hand when the feet are located at the focal plane distance from the sensors, the GOT method being the lowest performing one, well within the application margins with a value for the Dice coefficient of 0.966 at 800 mm.

While the performance of all methods is lower whenever the subject’s feet are outside the focal plane, all of them still produce correct results for the intended application. This is confirmed by the obtained Dice coefficients, being in all cases larger than 0.950 regardless of the distance; meanwhile, this distance does not differ in excess of 50 mm in any direction away from the focal plane.

Nonetheless, the reduced performance at distances outside the reference 800 mm mark highlights the importance of maintaining a working distance as close to the focal plane to keep an optimal registration between images acquired with the different cameras.

When analyzing each registration method performance, a method with larger degrees of freedom like the Homography does not necessarily achieve the best results, as it may introduce overfitting of the extra free parameters to compensate for errors in the registration not modelled by the underlying transformation model. Moreover, this method together with the GOT method, relies on the keypoints extracted from the checkerboard calibration images, limiting the accuracy of the registration mapping to fewer data points and increasing the impact of the keypoint detection errors into the registration results. These methods may benefit from checkerboards with a greater number of features, but the limited resolution of off-the-shelf thermal infrared cameras impose a restriction on how many features are accurately recognizable in the calibration process.

Additionally, the ICP and Affine-ASGD methods, which do not rely on checkerboard calibration, allow to correct possible drifts in the camera poses and optical characteristics changes during the prototype lifecycle without having to do a full re-calibration run with the checkerboard. Although these methods rely on the segmented images of the feet in the current application, this segmentation can be automated, this possibility being an important advantage over the methods that are not suitable for automation given their reliance on a calibration checkerboard.

## Figures and Tables

**Table 1:** Overlap performance with varying subject’s feet distance for different registration methods.

Metrics (Overlap Performance)	Focal Plane Distance (mm)	GOT	Homography	ICP	Affine-ASGD
Dice Coefficient	760	0.961	0.981	0.972	0.972
	780	0.966	0.980	0.978	0.978
	800	0.968	0.980	0.979	0.982
	820	0.965	0.971	0.975	0.979
	835	0.961	0.957	0.966	0.972
	850	0.953	0.949	0.959	0.966
Jaccard Index	760	0.925	0.963	0.946	0.946
	780	0.935	0.961	0.957	0.958
	800	0.938	0.961	0.959	0.965
	820	0.932	0.944	0.952	0.958
	835	0.924	0.918	0.933	0.945
	850	0.910	0.903	0.921	0.934
Volume Similarity	760	0.016	−0.002	0.003	−0.004
	780	0.020	0.004	0.010	0.000
	800	0.005	−0.009	−0.005	−0.014
	820	0.034	0.016	0.023	0.015
	835	0.013	0.002	0.006	−0.001
	850	0.016	0.004	0.006	−0.002
False Negative	760	0.031	0.020	0.026	0.025
	780	0.024	0.018	0.017	0.020
	800	0.030	0.024	0.024	0.022
	820	0.018	0.021	0.013	0.014
	835	0.033	0.042	0.031	0.033
	850	0.040	0.049	0.039	0.042
False Positive	760	0.047	0.018	0.029	0.022
	780	0.043	0.022	0.027	0.020
	800	0.034	0.016	0.019	0.008
	820	0.051	0.036	0.036	0.029
	835	0.046	0.044	0.037	0.031
	850	0.055	0.053	0.044	0.041

## Data Availability

The data presented in this study are available on request from the corresponding author. The data are not publicly available due to privacy restrictions.
